# Comparison of Adrenal Vein Sampling Methods for Subtyping of Primary Aldosteronism: A Retrospective Observational Study

**DOI:** 10.1007/s00270-025-04005-x

**Published:** 2025-04-02

**Authors:** Minfu Bai, Qiuping Zhao, Jiajia Dong, Xiaomo Yang, Xiaohui Wang, Chuanyu Gao

**Affiliations:** Department of Cardiology, Fuwai Central China Cardiovascular Hospital, Central China Fuwai Hospital of Zhengzhou University, 1 Fu Wai Road, Zhengzhou, 451464 China

**Keywords:** Primary aldosteronism, Adrenal venous sampling, Sampling methods in the adrenal vein

## Abstract

**Purpose:**

Although adrenal vein sampling (AVS) is the standard method for subtype diagnosis in patients with primary aldosteronism (PA), the methods used to sample the adrenal vein are not standardized. The aim of this study was to establish the optimal method for sampling the adrenal vein based on the pathological findings after surgery for PA.

**Methods:**

We enrolled 168 consecutive patients who were diagnosed to have PA and underwent AVS at our institution between 2019 and 2023. The impact of sampling by gentle negative pressure (GNP) on the accuracy of diagnosis of the PA subtype was compared with that of sampling by gravity, whereby blood flows out naturally.

**Results:**

AVS was performed successfully on both sides in 139 patients using the two sampling methods. Subtype diagnosis using the two sampling methods was concordant in 128 (92.1%) of the 139 patients and discordant in 11 (7.9%). Among the 11 patients with a discordant subtype diagnosis, unilateral adrenalectomy was performed in three with the right unilateral subtype by gravity and the bilateral subtype by GNP, one with the bilateral subtype by gravity and the right unilateral subtype by GNP, and one with the left unilateral subtype by gravity and the bilateral subtype by GNP. The pathological findings after surgery showed that the false-negative rate was 20% (1/5) with data obtained by the gravity method and 80% (4/5) with data obtained by the GNP method. Bilateral AVS took significantly longer when sampling was performed by the gravity method than when it was performed by GNP (*p* < 0.01).

**Conclusions:**

The gravity method may be preferable to GNP for AVS in patients with PA.

**Graphical Abstract:**

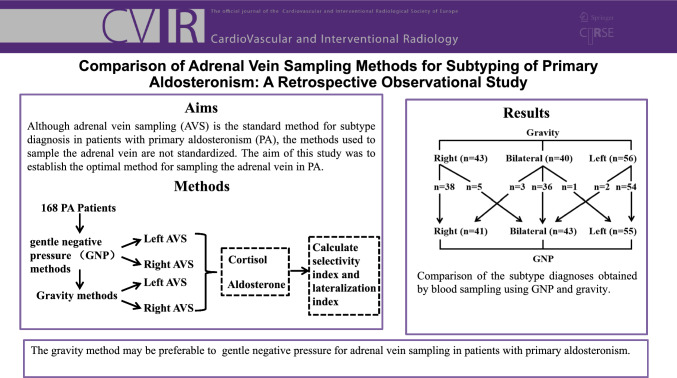

## Introduction

Adrenal vein sampling (AVS) is the international standard used to distinguish between unilateral and bilateral adrenal disease in patients with primary aldosteronism (PA), and unilateral secretion can be treated with surgery [1, 2]. Venous drainage from each adrenal gland occurs predominantly via a central vein. Recognition of the correct adrenal vein is essential when performing AVS. Selective cannulation of the right adrenal vein may be difficult because it is short, drains directly in the inferior vena cava (IVC), and often shares egress in the IVC with accessory hepatic veins. The training and experience of the operator are not seeming to fully account for the lower rate of successful cannulation on the right side [3]. The left adrenal vein emerges as a common trunk after joining the inferior phrenic vein and drains into the left renal vein such that selective catheterization can be consistently achieved on the left side [4].

Adrenal venous blood is rich in cortisol, which is used as a marker of successful AVS. The finding of a significantly higher cortisol level in an adrenal vein sample than in a peripheral sample vein confirms that the vein is draining mainly from the adrenal cortex. However, different sampling methods may produce different cortisol and aldosterone concentrations. Accurate sampling during AVS is mandatory for correct diagnosis and treatment of PA. We hypothesized that the sampling method used might impact the success rate of catheterization and judgment of the dominant secretory side. In addition, there is no information on the impact of the method used for AVS on the selectivity index and lateralization index. Therefore, this study was designed to prospectively investigate these questions by comparing the impact of sampling by gentle negative pressure (GNP) and that by gravity on the accuracy of diagnosis of the PA subtype.

## Methods Materials

### Study Design

The study had a single-center design and analyzed data for consecutive patients who underwent AVS at Fuwai Central China Cardiovascular Hospital between May 2019 and August 2023. Our department provides a tertiary referral service for patients with arterial hypertension. All patients were initially screened for PA by measurement of the plasma aldosterone to renin ratio. PA was diagnosed when this ratio was > 38 and confirmed by a saline infusion test and/or a captopril challenge test in accordance with the endocrine society guidelines [5]. The study was approved by the ethics committee at Fuwai Central China Cardiovascular Hospital.

### Adrenal Vein Sampling

All AVS procedures were performed by the same cardiologist, who had 8 years of experience in cardiovascular intervention at the start of the study period. Both adrenal glands were sampled simultaneously in all cases. Arenocorticotropic hormone (ACTH)-unstimulated AVS was performed throughout the procedure. After placing a catheter in each adrenal vein, blood was collected from both sides by gravity or GNP for measurement of the plasma cortisol concentration (PCC) and plasma aldosterone concentration (PAC). The steps performed for simultaneous AVS on both sides were as follows. First, a 5F MPA1 catheter (Cordis, Santa Clara, CA, USA) was used to locate and draw blood from the right adrenal vein via the median cubital vein pathway. The right adrenal vein orifice was identified by injection of a small amount of contrast medium. Next, a 5F SIM2 catheter (Cordis) was used to localize and draw blood from the left adrenal vein via the femoral vein route. Approximately 2 mL of residual contrast agent in the catheter was removed before collection of blood. A 5-mL syringe containing 2 mL of air was then used to simultaneously collect 3 mL of blood from the right and left adrenal veins by GNP, after which we collected 3 mL of blood from both sides using the gravity method. Patients were asked to avoid deep breathing during sampling to reduce the risk of displacement of the adrenal vein cannula in response to respiratory motion. We also injected a small amount of contrast medium to exclude the possibility of catheter displacement during blood sampling. Finally, 3 mL of blood were drawn from the IVC below the renal veins via the MPA1 catheter.

Successful catheterization was confirmed by measuring the cortisol ratio in the samples. Successful AVS was defined as a selectivity index of ≥ 2 for the levels of cortisol from each of the left and right adrenal veins to the level of cortisol from the IVC. The lateralization index was defined as a value of ≥ 2 when the aldosterone to cortisol (A/C) ratio on the dominant side was divided by the A/C ratio on the nondominant side [6, 7]. Unilateral adrenalectomy was performed in some patients with the unilateral subtype. The adrenocortical adenoma is positive for immunohistochemistry CD56, Melan-A, and Inhibin-α. The capsule is intact, and the reticular fiber staining is complete.

### Statistical Analysis

Continuous data are expressed as the mean ± standard deviation and were compared between groups using the paired-samples *t*-test if normally distributed and the Mann–Whitney *U* test if not normally distributed. Categorical data are shown as the frequency and percentage. Categorical variables were examined using the Chi-squared test or Fisher’s exact test. The concordance rate for subtype diagnosis was assessed using the Cohen´s Kappa test. All statistical analyses were performed using SPSS software (version 17.0; SPSS Inc., Chicago, IL, USA). All tests were two-tailed, and a *p*-value < 0.05 was considered statistically significant.

## Results

A total of 168 consecutive patients underwent AVS at our hospital during the study period. AVS was unsuccessful on the right side in 14 patients, on the left side in eight patients, and on both sides in six patients. In eight patients, AVS on the right was unsuccessful by GNP and successful by gravity. In five patients, AVS on the left was unsuccessful by GNP and successful by gravity. AVS was successful on both sides by the two sampling methods in 139 patients. Therefore, 147 patients by gravity sampling and 139 patients by GNP were successful on the right AVS. One hundred and forty-four patients by gravity sampling and 139 patients by GNP were successful on the left AVS.

**Fig. 1:**
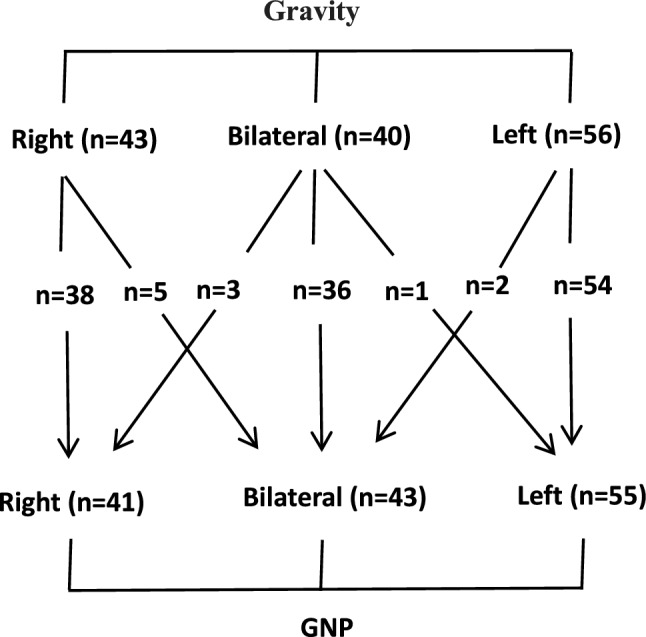
Comparison of the subtype diagnoses obtained by blood sampling using GNP and gravity. Ninety-nine patients were diagnosed with unilateral disease, and 40 patients were diagnosed with bilateral disease using the gravity sampling method. Of the 99 patients with unilateral disease diagnosed by the gravity sampling method, seven were diagnosed as having bilateral disease by the GNP sampling method. Four of the 40 patients diagnosed with bilateral disease using the gravity sampling method were diagnosed as having unilateral disease using the GNP sampling method. GNP, gentle negative pressure

Table 1 compares the results of AVS by GNP and by gravity. There was no significant difference in the PCC, PAC, A/C ratio, or the selectivity index on both sides for the adrenal vein on either side between the two sampling methods. The time required to perform AVS on both sides was significantly longer with the gravity method than with the GNP method (*p* < 0.01). The PA subtypes diagnosed by the two sampling methods are shown in Fig. 1. Sampling by the gravity method diagnosed the right unilateral subtype in 43 patients, the bilateral subtype in 40, and the left unilateral subtype in 56, while sampling by GNP diagnosed the right unilateral subtype in 41 patients, the bilateral subtype in 43, and the left unilateral subtype in 55.

**Table 1:** Comparison between the GNP and gravity sampling methods

Measurement		GNP	Gravity	*P*
PAC (pg/ml)	R	19,036.68 ± 60,865	18,587.83 ± 56,556	0.854
	L	6747.76 ± 12,017	8477.06 ± 16,474	0.168
PCC (pg/ml)	R	341.18 ± 349	348.13 ± 331	0.567
	L	183.74	208.73	0.229
A/C ratio	R	82.75 ± 254.05	65.18 ± 168.90	0.250
	L	58.97 ± 88.07	58.07 ± 82.23	0.781
SI	R	22.65 ± 11.65	21.87 ± 11.48	0.408
	L	14.36 ± 16.68	17.11 ± 24.03	0.102
Right adrenal vein (seconds)		61.13 ± 15.69	114.02 ± 23.11	< 0.001
Left adrenal vein (seconds)		37.56 ± 8.21	90.67 ± 10.95	< 0.001

The data are shown as the mean ± standard deviation or as the number

A/C ratio, aldosterone to cortisol ratio; B, bilateral; GNP, gentle negative pressure; L, left adrenal vein; LI, lateralization index; PAC, plasma aldosterone concentration; PCC, plasma cortisol concentration; R, right adrenal vein

Table 2 presented clinical and biochemical parameters in patients with discordant and concordant diagnoses between the two sampling methods. Diagnosis of PA subtype was concordant between the two sampling methods in 128 (92.1%) of the 139 patients and discordant in 11 (7.9%). The concordance rate for subtype diagnosis between the two methods was statistically significant (*к* = 0.88, *p* < 0.001). There were no significant differences in clinical or biochemical parameters according to whether the subtype diagnoses made by the two sampling methods were concordant or discordant.

**Table 2:** Comparison of clinical and biochemical parameters in patients with discordant and concordant diagnoses between the two sampling methods

Parameters	Discordant (*n* = 11)	Concordant (*n* = 128)	*P*
Age (years)	43.90 ± 7.05	46.43 ± 11.19	0.079
Sex, female (%)	60	54	0.748
Height, cm	167.20 ± 8.33	164.87 ± 7.83	0.809
Weight, kg	79.30 ± 17.71	70.59 ± 12.60	0.330
Course, months	66.40 ± 63.86	58.21 ± 77.16	0.832
HT drugs (number)	1.10 ± 1.20	1.71 ± 1.27	0.411
Serum K (mmol/l)	3.70 ± 0.48	3.34 ± 0.71	0.249
Serum creatinine, μmol/L	68.80 ± 26.78	65.29 ± 13.01	0.147
PAC (pg/ml)	317.11 ± 124.76	389.74 ± 311.25	0.072
PRA (pg/ml)	4.78 ± 3.49	5.20 ± 10.93	0.597
ARR	120.67 ± 126.79	224.91 ± 536.74	0.479

The data are shown as the mean ± standard deviation or as the number

ARR, aldosterone to renin ratio; HT, hypertension; PAC, plasma aldosterone concentration; PRA, plasma renin concentration

The subtype diagnosed by the two sampling methods was discordant in 11 patients. Two of these patients who were diagnosed to have the left unilateral subtype and five who were diagnosed to have the right unilateral subtype by gravity were found to have the bilateral subtype by GNP, three who were diagnosed to have the bilateral subtype by gravity were found to have the right unilateral subtype by GNP, and one diagnosed to have the bilateral subtype by gravity was diagnosed to have the left unilateral subtype by GNP. Table 3 shows the sex, age, subtype diagnosis, whether or not an adrenal nodule was detected on CT, and choice of treatment in the 11 patients with a discordant diagnosis. Unilateral adrenalectomy was performed in three patients with the right unilateral subtype by gravity and the bilateral subtype by GNP, one patient with the bilateral subtype by gravity and the right unilateral subtype by GNP, and one with the left unilateral subtype by gravity and the bilateral subtype by GNP. The remaining six patients were treated with a mineralocorticoid antagonist. The pathological findings after surgery confirmed a false-negative rate of 20% (1/5) for data obtained by the gravity method and 80% (4/5) for data obtained by the GNP method.

**Table 3:** Subtype and choice of treatment in 11 patients with discordant diagnoses

Case	Gender	Age, years	Subtype diagnosis						Adrenal nodule on CT	Treatment
			GNP (RSI)	Gravity (RSI)	GNP (LSI)	Gravity (LSI)	Gravity (LI)	GNP (LI)		
1	Female	51	35	36	20	23	R (4.2)	B (1.6)	None	R-ADR
2	Female	38	7	18	4	6	L (5.4)	B (1.4)	None	L-ADR
3	Male	42	49	51	31	26	B (1.2)	R (4.3)	R	R-ADR
4	Male	51	6	10	12	10	B (1.8)	R (5.2)	L	MRA
5	Male	38	35	25	4	4	R (3.3)	B (1.5)	L	MRA
6	Female	50	29	26	7	11	R (3.2)	B (1.1)	R	R-ADR
7	Male	46	28	33	18	11	R (5.1)	B (1.9)	R	R-ADR
8	Female	32	39	39	36	46	R (2.8)	B (1.8)	None	MRA
9	Female	39	10	11	102	153	B (1.2)	L (4.3)	L	MRA
10	Female	46	12	10	16	26	L (3.4)	B (1.2)	L	MRA
11	Male	55	7	9	12	10	B (1.8)	R (2.6)	R	MRA

ADR, adrenalectomy; B, bilateral; CT, computed tomography; GNP, gentle negative pressure; L, left; LI, lateralization index; LSI, left adrenal vein selectivity index; MRA, mineralocorticoid antagonist; R, right; RSI, right adrenal vein selectivity index

## Discussion

Although AVS is recognized as the optimal procedure for subtype diagnosis before adrenalectomy in patients with PA (9), there is no universal standard sampling method. Differences in the method used to take blood samples from the adrenal vein may produce differences in the PCC and PAC and affect subtype diagnosis. Previous studies have overlooked the effects of the sampling method used [8, 9] and collected blood for measurement of PCC and PAC from both sides by gravity or, if necessary, by GNP. The findings of this study indicate that the sampling method may impact on the diagnostic performance of AVS. We found no significant difference in the PCC, PAC, A/C ratio, or selectivity index between the two sampling methods for the right adrenal vein. However, in eight patients, AVS from the right adrenal vein was unsuccessful by GNP but successful by gravity. A possible explanation for this finding is that the right central adrenal vein is short, very small in caliber, drains directly into the posterior wall of the IVC, and often has an angulated path, causing the catheter tip to impact the intima and making it difficult to aspirate blood [9]. Therefore, in some patients, blood can flow out naturally by gravity from the catheter but cannot be collected by GNP.

The left adrenal vein emerges as a common trunk after joining the inferior phrenic vein and drains into the left renal vein. AVS from the common trunk of the left adrenal vein may be preferable in patients with PA [10]. In this study, we chose the common trunk as a sampling position and found no significant difference in the PCC, PAC, A/C ratio, or selectivity index between the two sampling methods when AVS was successful; however, in five patients, AVS of the left adrenal vein was unsuccessful by GNP but successful by gravity. Too rapid sampling by GNP makes it easy to draw more blood from the inferior phrenic vein and the renal vein, so the selectivity index may be less than the cutoff value.

Analysis of pathological outcomes in the patients with a discordant diagnosis showed that the subtype diagnosis might be more accurate when the gravity sampling method is used. There was discordance in subtype diagnosis between the two sampling methods in 11 patients. Four patients diagnosed as having the unilateral subtype by the gravity method and the bilateral subtype by the GNP method underwent unilateral adrenalectomy. These findings suggest that diagnosis of PA subtype could be more reliable when the gravity method is used and that subtype diagnosis by GNP may lead to false-negative results. The mechanism responsible for the false negatives obtained when the sampling method was GNP is unknown.

Disadvantages of the methods used to collect blood for AVS should also be considered. The disadvantage of the gravity method is that the time required to sample both adrenal veins is longer than that needed when using the GNP method.

This study has some limitations. First, it had a retrospective design and used AVS with concomitant data in the two sampling methods, which may have resulted in selection bias. Second, it is not clear whether our results are also applicable to AVS protocols with adrenocorticotropic hormone stimulation. Further studies in a larger number of cases are required to validate our findings. Potential bias could be overcome by increasing the number of patients with discordant data. Different results may be obtained with different catheters.

## Conclusion

The findings of this study indicate that subtype diagnosis is concordant between the gravity sampling method and the GNP sampling method in most patients with PA. Furthermore, false-negative subtype diagnoses might be less common with gravity sampling than with GNP sampling. The gravity method may be preferable for AVS in patients with PA. However, there is a need for additional studies in a larger number of cases with discordant results.
